# Role of population and test characteristics in antigen-based SARS-CoV-2 diagnosis, Czechia, August to November 2021

**DOI:** 10.2807/1560-7917.ES.2022.27.33.2200070

**Published:** 2022-08-18

**Authors:** Tomáš Kliegr, Jiří Jarkovský, Helena Jiřincová, Jaroslav Kuchař, Tomáš Karel, Ruth Tachezy

**Affiliations:** 1 Department of Information and Knowledge Engineering, Faculty of Informatics and Statistics, Prague University of Economics and Business, Prague, Czechia; 2 Institute of Health Information and Statistics of the Czech Republic, Prague, Czechia; 3 National Reference Laboratory for Influenza and Respiratory Viruses, National Institute of Public Health, Prague, Czechia; 4 Department of Software Engineering, Faculty of Information Technology, Czech Technical University in Prague, Czechia; 5 Department of Statistics and Probability, Faculty of Informatics and Statistics, Prague University of Economics and Business, Prague, Czechia; 6 Department of Genetics and Microbiology, Faculty of Science-BIOCEV, Charles University, Vestec, Czechia

**Keywords:** clinical evaluation, national study, SARS-Cov-2, rapid antigen test

## Abstract

**Background:**

Analyses of diagnostic performance of SARS-CoV-2 antigen rapid diagnostic tests (AG-RDTs) based on long-term data, population subgroups and many AG-RDT types are scarce.

**Aim:**

We aimed to analyse sensitivity and specificity of AG-RDTs for subgroups based on age, incidence, sample type, reason for test, symptoms, vaccination status and the AG-RDT’s presence on approved lists.

**Methods:**

We included AG-RDT results registered in Czechia’s Information System for Infectious Diseases between August and November 2021. Subpopulations were analysed based on 346,000 test results for which a confirmatory PCR test was recorded ≤ 3 days after the AG-RDT; 38 AG-RDTs with more than 100 PCR-positive and 300 PCR-negative samples were individually evaluated.

**Results:**

Average sensitivity and specificity were 72.4% and 96.7%, respectively. We recorded lower sensitivity for age groups 0–12 (65.5%) and 13–18 years (65.3%). The sensitivity level rose with increasing SARS-CoV-2 incidence from 66.0% to 76.7%. Nasopharyngeal samples had the highest sensitivity and saliva the lowest. Sensitivity for preventive reasons was 63.6% vs 86.1% when testing for suspected infection. Sensitivity was 84.8% when one or more symptoms were reported compared with 57.1% for no symptoms. Vaccination was associated with a 4.2% higher sensitivity. Significantly higher sensitivity levels pertained to AG-RDTs on the World Health Organization Emergency Use List (WHO EUL), European Union Common List and the list of the United Kingdom’s Department of Health and Social Care.

**Conclusion:**

AG-RDTs from approved lists should be considered, especially in situations associated with lower viral load. Results are limited to SARS-CoV-2 delta variant.

**Public Health impact of this article**

**What did you want to address in this study?**

It is important to know the sensitivity and specificity of SARS-CoV-2 tests, i.e. how well they recognise the infection even when the viral load is low and the person is asymptomatic. Although PCR should be preferred for SARS-CoV-2 testing, antigen tests are also often used. We therefore analysed nearly 350,000 paired antigen and PCR test results to determine which antigen tests work well and when.

**What have we learnt from this study? **

Test sensitivity was lower for children and adolescents, vaccinated individuals, saliva tests, tests conducted for preventive reasons and in periods of low SARS-CoV-2 incidence. Tests approved on the lists from the World Health Organization, the European Union and the UK Department of Health performed better.

**What are the implications of your findings for public health?**

There are considerable variations in the performance of antigen tests from different manufacturers. Only nasopharyngeal/nasal swabs, not saliva, should be used with antigen tests. Only antigen tests presented on the approved lists should be used, especially for children and adolescents.

## Introduction

Prior research has shown that there are marked differences between the sensitivity levels declared by the manufacturer and those observed in independent studies for antigen-detecting rapid diagnostic tests (AG-RDT) for severe acute respiratory syndrome coronavirus 2 (SARS-CoV-2) [1,2]. While there are multiple independent studies on AG-RDT performance, their utility is often limited because of small sample size, focus on a small number of AG-RDTs or use of analytical (in vitro) methods that have been reported to deviate from clinical evaluations [3]. Furthermore, there is a scarcity of evaluations of the diagnostic performance for population subgroups based on age, sampling method, symptoms and the level of SARS-CoV-2 incidence in the population.

For example, some results support the use of AG-RDTs for population screening [4,5], while other research suggests that during low SARS-CoV-2 incidence, false-positive results can lead to unnecessary economic costs [6]. One solution is to ensure that testing stops before the harm outweighs the benefits [3]. However, the necessary clinical studies determining the performance of AG-RDTs at various incidence levels are missing, which we attribute to the unavailability of sufficiently large data sets on AG-RDT use. To inform procurement of AG-RDTs, public bodies have compiled lists of approved AG-RDTs. However, to our knowledge, there are no studies evaluating the impact of using AG-RDTs from such lists on diagnostic performance in large population-based testing including specific subgroups such as children or asymptomatic individuals.

In this study, we compared the diagnostic performance of AG-RDTs using a unified methodology on data collected as part of the national testing efforts carried out in Czechia.

## Methods

### Cohort and data

The data were taken from the Czech Information System for Infectious Diseases (ISIN) with restriction of the date of the AG-RDT to the period between 5 August and 6 December 2021. The data had been inserted into ISIN in accordance with the country-level AG-RDT and PCR testing policies described below.

#### Indication for AG-RDT

There were four recorded reasons for referring a person to take a SARS-CoV-2 AG-RDT test as defined by the Ministry of Health of the Czech Republic [7]: The *diagnostic* indication was used for patients with symptoms. The *epidemiological* indication was used for contacts of confirmed cases or during contact tracing. The *preventive* indication was used for testing of individuals at risk, critical infrastructure of the state, mass screening as part of community testing and for testing of asymptomatic individuals. The *other* indication was also used but we did not analyse this separately as it was attributed to less than 1% of samples.

#### AG-RDT and PCR data collection

Results of AG-RDTs were inserted into ISIN following testing done by the primary state-guaranteed network of sampling sites and in the secondary network of healthcare providers in their offices, such as general practitioners, ambulatory specialists, dentists. Sampling sites could be run only by qualified healthcare providers and could use only tests approved for the Czech or European market [8]. A person with a positive AG-RDT would typically be referred for a confirmatory PCR test. 

Results of RT-PCRs were inserted into ISIN by a large number of laboratories using RT-PCR assays (further referred to as PCR) from different manufacturers. Laboratories performing SARS-CoV-2 testing by PCR had to successfully pass the external quality control programme (EQAP). Any reliable EQAP could be used, the WHO External Quality Assessment Project at the subnational level in 2021, the EQAP panel prepared by the Czech national reference laboratory for influenza and respiratory viruses, or any international EQAP programme managed by Quality Control for Molecular Diagnostics (QCMD) (QCMD) or INSTAND’s external quality assessment scheme (EQAS). Within EQAP, simulated samples with live or inactivated virus of different viral load were distributed. The concentration of viral genetic material corresponded to the concentration in a typical clinical sample [9]. Only laboratories that met the criteria and passed EQAP were accredited for PCR detection and thus only valid laboratory results were recorded to ISIN. However, PCR Cq values were not recorded in ISIN and thus Cq values were not available for this analysis.

#### Included AG-RDTs

Before analysis, we unified the names of AG-RDTs which the personnel in the network of testing sites had entered into ISIN. After this unification, which for some tests included merging several test versions from the same manufacturer, there were 467 distinct test types, although the actual number was probably smaller as some records contained information insufficient for merging (e.g. just the name of the manufacturer). For 16.1% of the AG-RDT results in the final dataset, no test identification (AG-RDT name or manufacturer) was available. The tests without test identification were included for subgroup analyses (such as by age), since these did not require identification of the test but were excluded from analyses of individual AG-RDTs. We estimate the number of unique AG-RDTs at 450. To ensure that the results reported for individual AG-RDTs were sufficiently statistically robust, we adopted from the latest release of the methodology used to compile the EU Common List (further referred to as the *EU Common List Guidelines*) the requirement of at least 100 PCR-positive and 300 PCR-negative samples per individual evaluated AG-RDT [10]. There were 38 matching AG-RDTs.

#### Determination of the sample type used with AG-RDT

The sample type was not included when the test results were inserted into ISIN. We used two methods to determine the sampling type for each test. The first method was based on the name of the AG-RDT. This could be done automatically for all AG-RDT types. We considered all tests whose names contained the word 'saliva' as saliva tests, all tests whose names included the word 'nasal' as nasal tests and all tests whose names included the word 'nasopharyngeal' as nasopharyngeal tests. Among these, 10 tests with multiple uses indicated in test name by the presence of a slash, such as in 'saliva/nasal', were excluded from the analyses of the effect of sample type. 

The second method used sampling type from the paired record in the EU database of COVID-19 in vitro diagnostic devices [11], which applied to 37 of the 38 AG-RDTs individually evaluated in our study. Out of these, we identified AG-RDTs for which one of the following sampling types was designated in the database: ‘saliva’, ‘nasal swab’, ‘nasopharyngeal swab’, or ‘nasal swab, nasopharyngeal swab’. Tests with other sampling types or other combinations were excluded for the analyses of sample type. The list of AG-RDTs according to the EU database and the list according to test name are provided in the Supplement. 

Both methods were applied independently, and their results were compared. 

#### Information on age, vaccination status and indication

Age information was available for more than 99.99% of the samples (i.e. all except for 10). We defined four age subgroups (boundaries are inclusive): children (0–12 years), adolescents (13–18 years), young adults (19–25 years) and other adults (≥ 26 years). Samples were also associated with information on vaccination status. Persons were considered vaccinated at the time of the AG-RDT if they were at least 2 weeks after the first dose of the Janssen Ad26.COV2.S COVID-19 vaccine or 2 weeks after a second dose of the other vaccines.

#### Characterisation of viral load and symptoms

Since Cq values were not recorded in ISIN, the analysed dataset did not contain information on viral load. However, we correlated viral load with symptoms, which were available in ISIN. Asymptomatic individuals are reported in the literature to have Cq values between 27.2 and 30.5 and symptomatic individuals typically between 20.5 and 27.0 [1]. Symptoms were coded in ISIN as binary indicators of 'cough', 'muscle ache/fever', 'diarrhoea/vomiting', 'loss of smell/taste' or 'other symptoms'. A person was considered symptomatic in our dataset if they were reported to exhibit at least one symptom.

#### Determination of incidence by date and region

For more than 98% of samples in the dataset, the date of the AG-RDT was available, as well as the administrative region ('kraj' in Czech) in which the test was performed. Samples without the region information were excluded. With the combination of date and region, we inferred the number of newly infected persons per 100,000 in the preceding 7-day period (this value is further referred to as *incidence*). The incidence was sourced from an open dataset published by the Czech Ministry of Health [12].

#### Lists of approved AG-RDTs

The versions of the lists we used were:

- World Health Organization (WHO): *Emergency use listing for in vitro diagnostics (IVDs) detecting SARS-CoV-2* (version from 2 October 2020) [13],
- World Health Organization (WHO): *Emergency use listing for in vitro diagnostics (IVDs) detecting SARS-CoV-2* (last update on 7 June 2022) [14],
- European Union (EU): *A common list of COVID-19 rapid antigen tests* (6 May 2022) [10],
- United Kingdom (UK) Department of Health and Social Care (DHSC): *Outcome of the evaluation of rapid diagnostic assays for specific SARS-CoV-2 antigens* (6 June 2022) [15].
- Germany – Paul Ehrlich Institute (PEI):- *Results of the comparative evaluation of the sensitivity of SARS-CoV-2 antigen rapid tests* (30 May 2022) [16].

For the WHO list, we also included an older, 2020 version of the list. This was done to represent information available at the time of the assumed procurement of the AG-RDTs used in the studied period. The methodologies used to compile these lists differ substantially. The WHO Emergency Use List (EUL) and the EU Common List are based on analysis of secondary evidence, including that provided by the manufacturer and independent validation studies. The two other included lists are based on evaluations performed by the respective national agencies. 

The UK DHSC commissioned an independent evaluation of AG-RDTs. In Phase 1, a desktop review was performed to remove tests from further evaluation based on factors such as early product stage or low manufacturing capacity. Phase 2 of constructing the UK DHSC list was aimed at determining performance with spiked samples, including evaluation of cross-reactivity. About 30% of the highest performing AG-RDTs passed to Phase 3, where each test was evaluated against at least 200 frozen positive samples and 1,000 fresh true-negative samples [4,17]. 

The PEI in Germany used smaller sets of samples to determine AG-RDT sensitivities [18,19] and evaluated individual AG-RDTs using 50 frozen pools of hundreds of nasopharyngeal and oropharyngeal swabs from SARS-CoV-2-positive individuals; the subgroup analyses (three ranges of Cq values) were based on between nine and 23 pools. A test passed with PEI if it had at least 75% sensitivity for high viral load samples. Since the PEI list was the only one that published concrete sensitivities for all AG-RDTs, we used it to compute the average sensitivity of all ca 250 AG-RDTs on the PEI list and compared this number with the average sensitivity obtained in our study. From the PEI study, we used sensitivities for medium (Cq > 25 – < 30) and high viral load (Cq ≤ 25). The sensitivity levels for low viral load (Cq ≥ 30) equalled zero for most tests on the PEI lists and therefore, we did not use this level from the PEI study.

#### Pairing of AG-RDT and PCR tests

Our analysis was based on a group of persons for whom an AG-RDT test was taken up to 3 days before the PCR test and the results for both tests were registered to ISIN. Through the 3-day interval, we could extend the dataset size because taking a confirmatory PCR after AG-RDT often required registration associated with 1 day or more of waiting time. According to the EU Common List Guidelines, clinical studies should evaluate diagnostic performance on unselected samples taken from symptomatic and asymptomatic participants that should represent naturally occurring viral loads. By limiting our analysis to AG-RDT and PCR performed on the same day, we would over-represent cases with a higher probability of infection and symptoms (including those not reported) indicative of taking AG-RDT and PCR on the same day. We analysed the effect of various delays up to 3 days, and the 3-day cut-off resulted in the highest match with prior evaluations. For individuals reporting symptoms, the sensitivity of tests on the WHO 2020 list, when compared against PCR performed 3 days after the AG-RDT, was 81.3%, suggesting that at least for cases with a higher viral load, which is statistically associated with symptoms [1], the 3-day interval is not too long.

### Performed types of analyses

#### Evaluation of individual AG-RDTs

We separately report on 38 AG-RDTs for which there were at least 100 PCR-positive and 300 PCR-negative samples. We also included two figures comparing AG-RDTs in the different age groups and the SARS-CoV-2 incidence per 100,000 inhabitants in the administrative region where the AG-RDT was performed. In these comparisons, we included only that subset of the 38 AG-RDTs for which there were at least 50 PCR-positive and 50 PCR-negative samples for each age and incidence group.

#### Analyses of subgroups

Subgroup analyses for sample types as determined from the test name were performed on the complete dataset of ca 450 AG-RDTs, as were the analyses of the effect of the number of days between AG-RDT and PCR test, age, incidence level and vaccination status. The subgroup analysis for sample types based on the metadata from the EU database was performed on the subset of the 38 most commonly used tests. This subset was also used for analysing the effect of an AG-RDT being included in the WHO EUL, the EU Common List, the UK DHSC list or the PEI list.

#### Measures of diagnostic performance and statistical analysis

As the primary measure, we adopted clinical sensitivity and clinical specificity. Let *TP* denote the number of samples that were PCR-positive and also AG-RDT positive, *FN* the number of samples that were PCR-positive and AG-RDT negative, *FP* the number of samples that were PCR-negative and AG-RDT positive, and *TN* the number of samples that were PCR-negative and AG-RDT negative. The sensitivity values were computed as *TP*/(*TP* + *FN*), and the specificity values as *TN*/(*TN* + *FP*). The positive predictive value (PPV) was calculated as 1 − (*FP*/(*TP* + *FP*)) and the negative predictive value (NPV) as *TN*/(*TN* + *FN*). We calculate 95% confidence intervals (CI) (presented as error bars in figures) using Wald's continuity correction [20]. Note that, if CI of two means were not overlapping, the null hypothesis of the equality of two means was rejected [21]. For validation of statistical significance of difference in sensitivities, we used a one-sided 95% exact Clopper–Pearson CI for the binomial distribution [22]. Where this resulted in a statistical significance level below 5%, this is denoted as p < 0.05. To calculate correlations between our sensitivities and those reported by PEI, we use the Pearson correlation coefficient [23].

## Results

In the study period, the Delta SARS-CoV-2 mutation was dominant in Czechia (www.gisaid.org). Our dataset consisted of 346,221 AG-RDT results for which a PCR test was performed within 3 days of the AG-RDT. About 450 different types of AG-RDT were used; the average AG-RDT positivity level was 17.0% and the average PCR positivity level was 19.8%. There were 49,618 true positives, 9,111 false positives, 18,961 false negatives and 268,531 true negatives. The average AG-RDT sensitivity was 72.4% (n = 346,221; 95% CI: 72.0–72.7), and the average specificity was 96.7%.

### Evaluation of individual AG-RDTs

The diagnostic performance of the 38 investigated tests is presented in Table 1. Sensitivities of the tests varied between 18.4% for no. 16 (Realy Tech Saliva) to 92.7% for no. 31 (SD Biosensor Standard Q). The specificity values ranged from 90.0% to 99.9%. These 38 AG-RDTs accounted for ca 78.5% (272,000) of the total 346,221 pairs of AG-RDT and PCR samples in the studied dataset.

**Table 1:** Evaluation of SARS-CoV-2 antigen rapid diagnostic tests, Czechia, 5 August–6 December 2021 (n = 38 tests, n = 272,000 samples)

ID	Test name	Manufacturer	Total samples	PCR-positive cases	Sensitivity in % (95% CI)	Specificity in % (95% CI)
AG-RDTs with at least 100 PCR-positive and 300 PCR-negative cases						
1	ANTIGEN RAPID TEST CASSETTE SARS-COV-2 (SWAB)	A. Menarini Diagnostics	1,685	253	55.7 (49.2–62.2)	98.4 (97.7–99.1)
2	Panbio Covid-19 Ag Rapid Test	Abbott Rapid Diagnostics	26,949	6,861	79.9 (78.9–80.8)	97.1 (96.8–97.3)
3	Flowflex SARS-CoV-2 Antigen rapid test	ACON Biotech (Hangzhou) Co., Ltd.	6,825	1,949	85.4 (83.8–87.0)	95.3 (94.7–95.9)
4	SARS-CoV-2 Antigen Rapid Test (Nasal/Saliva)	ACON Biotech (Hangzhou) Co., Ltd.	577	200	77.5 (71.2–83.8)	93.4 (90.6–96.1)
5	ECOTEST COVID-19 Antigen Rapid Test Device	Assure Tech (Hangzhou) Co., Ltd.	434	116	82.8 (75.0–90.5)	92.8 (89.6–95.9)
6	AMP Rapid Test SARS-CoV-2 Ag	AMEDA Labordiagnostic GmbH	1,169	273	78.8 (73.5–84.0)	94.8 (93.2–96.3)
7	SARS-CoV-2 Antigen Rapid Test Kit	Beijing Lepu Medical Technology Co., Ltd.	49,505	6,720	48.7 (47.5–49.9)	97.8 (97.7–98.0)
8	WANTAI SARS-COV-2 Ag Rapid Test (Colloidal gold)	BEIJING WANTAI BIOLOGICAL PHARMACY ENTERPRISE CO. LTD	4,844	632	80.9 (77.6–84.1)	96.1 (95.5–96.7)
9	BIOSYNEX COVID-19 Ag BSS	BIOSYNEX S.A.	936	546	87.4 (84.4–90.3)	91.5 (88.5–94.6)
10	DIAQUICK COVID-19 Ag Cassette	DIALAB Ges.m.b.H, Wr.Neudorf/AT	1,795	316	81.0 (76.4–85.6)	96.7 (95.7–97.7)
11	EBS SARS-CoV-2 Ag Rapid Test	EUROBIO SCIENTIFIC	8,145	567	50.6 (46.3–54.9)	98.9 (98.7–99.2)
12	SARS-CoV-2 Antigen Test Kit (Colloidal Gold)	Genrui Biotech Inc.	1,044	213	61.0 (54.0–68.0)	97.4 (96.1–98.6)
13	V-CHEK, 2019-nCoV Ag Rapid Test Kit (Immunochromatography)	Guangzhou Decheng Biotechnology Co., LTD.	855	435	86.4 (83.0–89.9)	91.7 (88.8–94.5)
14	Wondfo 2019-nCoV Antigen Test (Lateral Flow Method)	Guangzhou Wondfo Biotech Co. Ltd	12,598	1,911	48.2 (46.0–50.5)	98.4 (98.2–98.7)
15	Novel Coronavirus (SARS-Cov-2) Antigen rapid test	Hangzhou Realy Tech Co., Ltd.	827	157	67.5 (59.6–75.5)	93.1 (91.1–95.2)
16	Novel Coronavirus (SARS-Cov-2) Antigen rapid test Device (saliva)	Hangzhou Realy Tech Co., Ltd.	2,639	266	18.4 (13.4–23.4)	98.5 (98.0–99.1)
17	Novel Coronavirus (SARS-Cov-2) Antigen Rapid Test Cassette (swab)	Hangzhou Realy Tech Co., Ltd.	3,230	620	66.0 (62.1–69.9)	97.7 (97.0–98.3)
18	COVID-19 Antigen Test Kit (Colloidal Gold)	Hangzhou Singclean Medical Products Co., Ltd.	3,044	651	65.0 (61.2–68.8)	97.2 (96.5–97.9)
19	Humasis COVID-19 Ag Test	HUMASIS Co. Ltd	19,290	5,289	77.5 (76.4–78.6)	97.2 (96.9–97.5)
20	SARS-CoV-2 Antigen Test Cassette (Nasal Swab Specimen)	Jiangsu Mole Bioscience Co., Ltd.	1,106	158	39.2 (31.0–47.5)	99.1 (98.3–99.8)
21	Saliva Orawell Covid_19 Ag	Jiangsu Well Biotech Co. Ltd	561	188	71.8 (64.9–78.8)	93.8 (91.1–96.5)
22	Wellion SARS-CoV-2 PLUS ANTIGEN Rapid Test	MED TRUST Handelsges.m.b.H.	2,595	1,190	86.4 (84.4–88.4)	93.5 (92.1–94.8)
23	NADAL COVID-19 Ag Test	nal von minden GmbH	22,818	4,802	82.0 (80.9–83.1)	96.3 (96.0–96.6)
24	COVID-19 Antigen Test Kit (Colloidal Gold)	Nantong Diagnos Biotechnology Co.,Ltd.	543	104	76.9 (67.9–86.0)	98.9 (97.6–100.0)
25	COVID-19 Antigen Detection Kit	New Gene (Hangzhou) Bioengineering Co., Ltd.	3,110	718	74.2 (70.9–77.6)	98.0 (97.4–98.6)
26	(SARS-CoV-2) Antigen Rapid Test COVIDENT COVID-19	Pierenkemper GmbH	684	193	49.7 (42.2–57.3)	96.1 (94.2–98.0)
27	SARS-CoV-2 Rapid Antigen Test	Roche (SD BIOSENSOR)	4,948	1,357	82.1 (80.0–84.2)	97.5 (96.9–98.0)
28	SARS-CoV-2 Rapid Antigen Test Nasal	Roche (SD BIOSENSOR)	643	289	87.5 (83.4–91.7)	94.1 (91.3–96.8)
29	COVID-19 Antigen Rapid Test Kit (Swab)	Safecare Biotech (Hangzhou) Co., Ltd.	18,318	3,080	55.0 (53.2–56.8)	96.7 (96.4–97.0)
30	STANDARD F COVID-19 Ag FIA	SD Biosensor, Inc	3,907	478	79.5 (75.7–83.3)	98.7 (98.3–99.1)
31	STANDARD Q COVID-19 Ag Test	SD Biosensor, Inc	579	192	92.7 (88.5–96.9)	95.3 (93.0–97.7)
32	SARS-CoV-2 Antigen Test Kit	Shenzhen Ultra-Diagnostics Biotec.Co.,Ltd	6,883	947	54.1 (50.8–57.3)	99.9 (99.8–100.0)
33	Rapid-VIDITEST COVID-19 Antigen	Vidia spol. s r.o.	5,139	507	25.6 (21.6–29.6)	97.5 (97.0–98.0)
34	ViVaDiag SARS CoV 2 Ag Rapid Test	VivaChek Biotech (Hangzhou) Co., Ltd.	11,099	2,847	79.1 (77.5–80.6)	95.5 (95.0–95.9)
35	VivaDiag Wellion SARS-CoV-2 Antigen Rapid Test	VivaChek Biotech (Hangzhou) Co., Ltd.	1,768	921	86.2 (83.9–88.5)	90.0 (87.8–92.1)
36	VivaDiagTM Pro SARS-CoV-2 Ag Rapid Test	VivaChek Biotech (Hangzhou) Co., Ltd.	34,696	5,967	75.8 (74.7–76.9)	97.0 (96.8–97.2)
37	SARS-CoV-2 Antigen Rapid Test Kit	WuHan UNscience Biotechnology Co., Ltd.	2,049	544	66.9 (62.8–71.0)	96.0 (95.0–97.1)
38	COVID-19 Antigen Detection Kit (Colloidal Gold)	ZHUHAI LITUO BIOTECHNOLOGY CO., LTD	4,163	764	62.8 (59.3–66.4)	96.3 (95.6–96.9)

AG-RDT: antigen rapid diagnostic test; CI: confidence interval; COVID-19: coronavirus disease; ID: identification number; SARS-CoV-2: severe acute respiratory syndrome coronavirus 2.

AG-RDTs are sorted by manufacturer name. For AG-RDTs 7, 13, 17, 18, 21 and 37, the entry includes an additional AG-RDT variant or variants as specified in the Supplement.

Data source: Czech Information System for Infectious Diseases.

### Comparison of AG-RDTs by age group, incidence, indication, symptoms and vaccination status

Data presented in Table 2 show the results of AG-RDT performance for selected subgroups. Sensitivity in children (0–12 years) and adolescents (13–18 years) was significantly lower than in adults (p < 0.05). The average diagnostic performance varied by SARS-CoV-2 incidence. The higher the incidence, the higher the sensitivity and the lower the specificity. We defined four incidence levels (7-day incidence per 100,000): < 100, ≥ 100 – < 500, ≥ 500 – < 1,000 and ≥ 1,000. The sensitivity for each of the first four incidence levels was statistically significantly lower than the sensitivity for the highest incidence category of ≥ 1,000 (p < 0.05). Significantly higher sensitivity was obtained for the diagnostic indication (suspected SARS-CoV-2 infection) compared with the epidemiological or preventive indications (p < 0.05). Significantly higher sensitivity levels were recorded when symptoms were present (p < 0.05). Since the AG-RDT test sensitivity level was unexpectedly about four percentage points higher for vaccinated than for unvaccinated persons (p < 0.05), we separately compared these subgroups based on the presence or absence of symptoms. Sensitivity levels for both vaccinated subgroups (symptomatic and asymptomatic) were also higher than for the corresponding unvaccinated subgroups (p < 0.05).

**Table 2:** Average performance of SARS-CoV-2 antigen rapid diagnostic tests across age groups and incidence levels, Czechia, 5 August–6 December 2021 (n = 450 tests, n = 346,211 samples)

Group	Total samples	PCR-positive cases		PCR test positivity in %	Sensitivity in % (95% CI)	Specificity in % (95% CI)	PPV in %	NPV in %
**Age (years), n = 346,211**								
0–12	44,896	5,489		12.2	65.5 (64.2–66.7)	97.0 (96.8–97.2)	75.2	95.3
13–18	37,693	5,101		13.5	65.3 (64.0–66.6)	97.2 (97.0–97.4)	78.6	94.7
19–25	37,126	6,153		16.6	71.0 (69.9–72.2)	97.0 (96.8–97.4)	82.6	94.4
≥ 26	226,496	51,835		22.9	73.9 (73.5–74.3)	96.5 (96.4–96.6)	86.3	92.6
**SARS-CoV-2 incidence (new cases per 100,000 persons in the preceding 7 days), n = 346,221**								
0–100	154,081	6,877		4.5	66.0 (64.8–67.1)	98.3 (98.3–98.4)	65.0	98.4
100–500	83,114	18,682		22.5	68.8 (68.1–69.4)	96.5 (96.4–96.7)	85.2	91.4
500–1,000	64,062	23,804		37.2	73.5 (72.9–74.1)	93.9 (93.7–94.2)	87.8	85.7
1,000–1,727	44,964	19,216		42.7	76.7 (76.1–77.3)	92.3 (91.9–92.6)	88.1	84.1
**Indication, n = 343,062**								
Diagnostic	45,039	22,423	49.8		86.1 (85.7–86.6)	91.6 (91.2–92.0)	91.1	87.0
Epidemiological	71,442	12,279	17.2		63.6 (62.8–64.5)	96.4 (96.3–96.6)	78.8	92.7
Preventive	226,581	311,61	13.8		63.6 (63.1–64.2)	97.5 (97.4–97.6)	80.3	94.4
**Symptoms, n = 346,221**								
No symptoms	229,701	28,605	12.5		57.1 (56.6–57.7)	97.6 (97.5–97.6)	77.1	94.1
At least one symptom	60,909	29,669	48.7		84.8 (84.4–85.2)	92.0 (91.7–92.3)	90.9	86.4
Symptom information missing	55,611	10,305	18.5		78.8 (78.0–79.6)	96.2 (96.0–96.3)	82.4	95.2
**Vaccination status, n = 346,221**								
Unvaccinated	235,795	42,985	18.2		70.8 (70.3–71.2)	96.9 (96.8–96.9)	83.4	93.7
No symptoms	164,478	18,859	11.5		55.8 (55.1–56.5)	97.6 (97.5–97.7)	75.3	94.5
At least one symptom	33,686	17,687	52.5		84.2 (83.7–84.8)	91.1 (90.6–91.5)	91.3	83.9
Vaccinated	110,426	25,594	23.2		75.0 (74.5–75.5)	96.4 (96.3–96.5)	86.2	92.7
No symptoms	65,223	9,746	14.9		59.7 (58.8–60.7)	97.5 (97.3–97.6)	80.5	93.2
At least one symptom	27,223	11,982	44.0		85.6 (85.0–86.2)	92.9 (92.5–93.3)	90.4	89.1

CI: confidence interval; NPV: negative predictive value; PPV: positive predictive value; SARS-CoV-2: severe acute respiratory syndrome coronavirus 2.

The total sample sizes for age and indication are smaller than the other totals because for 10 samples, information on age information was missing, and we excluded the indication subgroup ‘other’ as explained in the Methods section. For analysis of vaccination by symptoms, the sum of ‘No symptoms’ and ‘At least one symptom’ is lower than the corresponding subtotal for (un)vaccinated because the subgroups with missing symptoms are omitted.

Data source: Czech Information System for Infectious Diseases.

**Figure 1:**
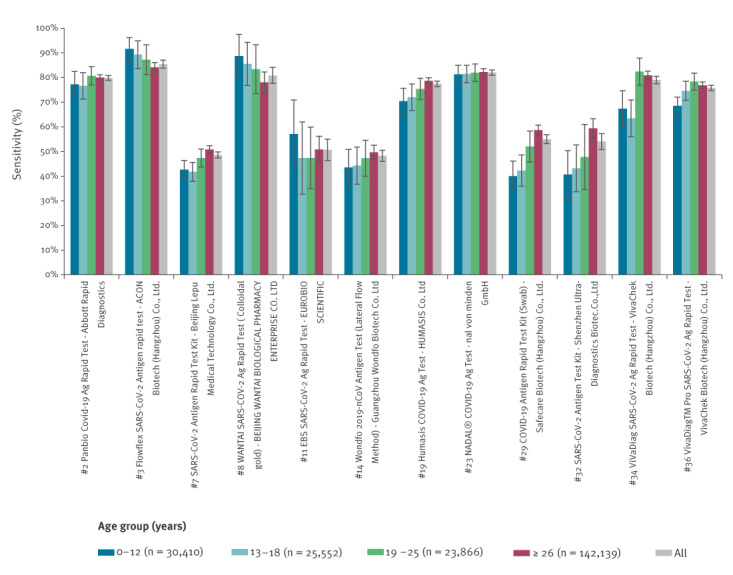
Sensitivity levels of SARS-CoV-2 antigen rapid diagnostic tests by age group, Czechia, 5 August–6 December 2021 (n = 12 tests; n = 221,967 samples)

**Figure 2:**
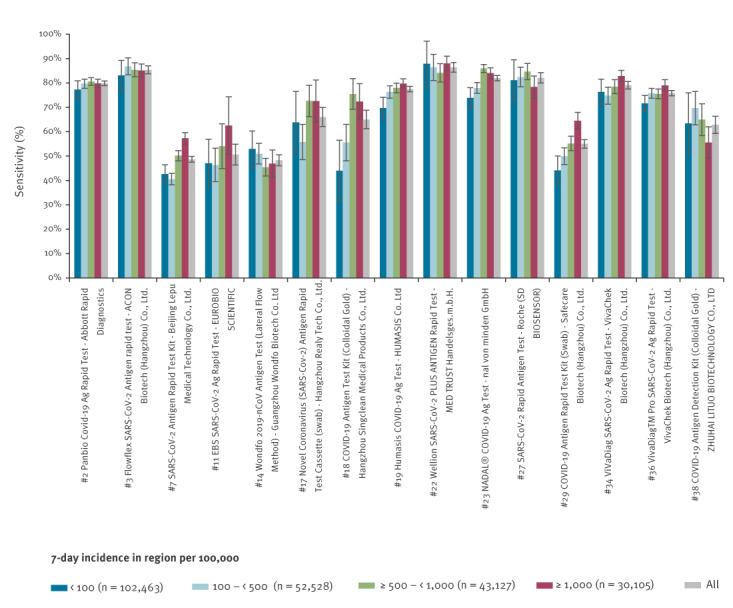
Sensitivity levels of SARS-CoV-2 antigen rapid diagnostic tests by incidence per 100,000 persons, Czechia, 5 August–6 December 2021 (n = 15 tests; n = 228,223 samples)

The sensitivity levels of individual AG-RDTs with respect to age and incidence are presented in Figures 1 and 2. Of the 12 tests shown in Figure 1, AG-RTDs no. 7, 29, 32 and 34 had a statistically significantly lower sensitivity (p < 0.05) in children and adolescents (0–18 years) compared with the largest group of adults (≥ 26 years). Sensitivity of test no. 36 was significantly lower in the subgroup aged 0–12 years compared with those 26 years and older (p < 0.05). The largest differences were recorded for the test manufactured by Safecare (no. 29), where the sensitivity in the youngest age group was 39.9% (n = 2,670; 95% CI: 33.7–46.0) compared with 58.7% (n = 11,154; 95% CI: 56.6–60.8) in adults 26 years and older.

SARS-CoV-2: severe acute respiratory syndrome coronavirus 2.

Included are all tests from Table 1 for which there were at least 50 PCR-positive and 50 PCR-negative cases in each of the age groups (n = 12).

Data source: Czech Information System for Infectious Diseases.

SARS-CoV-2: severe acute respiratory syndrome coronavirus 2.

Included are all tests from Table 1 for which there were at least 50 PCR-positive and 50 PCR-negative cases in each of the incidence levels (n = 15).

Data source: Czech Information System for Infectious Diseases.

Among the 15 tests shown in Figure 2, AG-RTDs no. 7, 18, 23 and 29 had a statistically significantly lower sensitivity (p < 0.05) in a low-incidence setting (< 500/100,000) than in a high-incidence setting (≥ 1,000/100,000). The largest difference in incidence was recorded for the test manufactured by Singclean (no. 18), which had at incidence < 100 per 100,000 a sensitivity of 44.0% (n = 1,441; 95% CI: 31.5–56.5) compared with 72.4% (n = 360; 95% CI: 65.1–79.7) at incidence ≥ 1,000 per 100,000.

### Evaluation of AG-RDTs based on sample type

In Table 3, we list the aggregate results for all tests according to the sample type. Note that, in the wider group of saliva tests as determined based on test name, two met the criteria of at least 100 positive PCR samples and 300 negative PCR samples and were thus included in Table 1 as no. 16 (Saliva test Realy Tech) and no. 21 (Saliva Orawell). The sensitivity of no. 16 was 18.4% (Table 1), which was the lowest value among all AG-RDT tests meeting the selection criteria. The table contains also a swab version by the same manufacturer with a sensitivity level of 66.0%. In contrast, the sensitivity level of 71.8% for no. 21 was close to the average sensitivity of 72.4% for the entire dataset.

**Table 3:** Performance of SARS-CoV-2 antigen rapid diagnostic tests based on sample type determined according to two methods: test name and EU database, Czechia, 5 August–6 December 2021

Group of AG-RDTs (distinct tests)	Total samples	PCR-positive cases	PCR test positivity in %	Sensitivity in % (95% CI)	Specificity in % (95% CI)	PPV in %	NPV in %
Sample type determined from test name (from all AG-RDT tests in the analysed dataset), n = 74 tests, n = 6,545 samples							
Saliva (n=36)	4,016	668	16.6	51.6 (47.7–55.6)	95.8 (95.1–96.5)	71.2	90.9
Nasal (n=24)	2,349	651	27.7	73.9 (70.4–77.4)	97.1 (96.2–97.9)	90.6	90.6
Nasopharyngeal (n=14)	180	70	38.9	84.3 (74.4–94.2)	89.1 (82.4–95.8)	83.1	89.9
Sample type determined from the EU database (from the 38 AG-RDT tests in Table 1), n = 21 tests, n = 118,617 samples							
Saliva (n=1)	2,639	266	10.1	18.4 (13.4–23.4)	98.5 (98.0–99.1)	58.3	91.5
Nasal swab (n=7)	35,313	5,522	15.6	58.6 (57.2–59.9)	97.3 (97.1–97.5)	80.3	92.7
Nasal swab, Nasopharyngeal swab (n=8)	67,751	16,932	25.0	78.7 (78.1–79.4)	97.1 (96.9–97.2)	89.9	93.2
Nasopharyngeal swab (n=5)	12,914	2,596	20.1	79.7 (78.1–81.3)	97.8 (97.5–98.1)	90.2	95.0

AG-RDT: antigen rapid diagnostic test; CI: confidence interval; EU: European Union; NPV: negative predictive value; PPV: positive predictive value; SARS-CoV-2: severe acute respiratory syndrome coronavirus 2.

The numbers in parentheses after the sample types indicate the number of matching AG-RDTs.

Data source: Czech Information System for Infectious Diseases.

### Effect of the number of days between AG-RDT and PCR test

Data presented in Table 4 shows that sensitivity decreased with increasing time between the AG-RDT and the PCR test. The second part of the table shows that the dynamics of this decrease in sensitivity depended on the presentation of clinical symptoms and on the presence of a particular AG-RDT on the list of AG-RTDs approved by the WHO (see next section).

**Table 4:** Data on SARS-CoV-2 antigen-detecting rapid diagnostic tests and subsequent PCR tests, Czechia, 5 August–6 December 2021 (n = 450 tests, n = 346,221 samples)

Number of days between AG-RDT and PCR	Total samples	PCR-positive cases	PCR test positivity in %	Sensitivity in % (95% CI)	Specificity in % (95% CI)	PPV in %	NPV in %
PCR up to 3 days after AG-RDT (n = 450 test types)							
0–3	346,221	68,579	19.8	72.4 (72.0–72.7)	96.7 (96.7–96.8)	84.5	93.4
Composite subdatasets (n = 450)							
0	86,016	15,945	18.5	80.4 (79.8–81.1)	96.0 (95.9–96.2)	82.2	95.6
1	140,265	31,421	22.4	80.1 (79.6–80.5)	95.9 (95.8–96.0)	84.9	94.3
2	60,758	12,971	21.3	64.6 (63.8–65.4)	97.3 (97.2–97.5)	86.8	91.0
3	59,182	8,242	13.9	39.6 (38.5–40.6)	98.9 (98.8–99.0)	85.2	91.0
AG-RDT on WHO list 2022 (n = 3), no symptoms reported							
0	6,392	1,057	16.5	84.8 (82.5–87.0)	97.0 (96.5–97.5)	84.8	97.0
1	9,020	1,471	16.3	78.2 (76.0–80.4)	97.6 (97.2–97.9)	86.2	95.8
2	3,254	494	15.2	52.8 (48.2–57.4)	98.7 (98.2–99.1)	87.6	92.1
3	3,786	409	10.8	24.2 (19.8–28.6)	99.1 (98.8–99.5)	76.7	91.5
AG-RDT not on WHO list 2022 (n = 35), no symptoms reported							
0	40,913	3,809	9.3	65.3 (63.8–66.9)	97.5 (97.3–97.6)	72.6	96.5
1	81,074	10,862	13.4	69.7 (68.8–70.6)	97.1 (97.0–97.2)	78.9	95.4
2	36,375	5,029	13.8	44.4 (43.0–45.8)	98.4 (98.2–98.5)	81.5	91.7
3	36,842	3,857	10.5	17.0 (15.7–18.2)	99.5 (99.4–99.6)	79.1	91.1
AG-RDT on WHO list 2022 (n = 3), symptoms reported							
0	3,668	1,586	43.2	87.2 (85.5–88.9)	94.7 (93.7–95.7)	92.6	90.7
1	5,598	2,648	47.3	89.7 (88.5–90.8)	93.5 (92.6–94.4)	92.6	91.0
2	1,726	926	53.7	89.0 (86.9–91.1)	89.5 (87.3–91.7)	90.7	87.5
3	909	411	45.2	81.3 (77.3–85.3)	91.6 (88.9–94.2)	88.8	85.6
AG-RDT not on WHO list 2022 (n = 35), symptoms reported							
0	11,344	4,748	41.9	84.4 (83.3–85.4)	93.0 (92.3–93.6)	89.6	89.2
1	18,714	9,859	52.7	86.8 (86.1–87.5)	91.1 (90.5–91.7)	91.6	86.1
2	7,816	4,135	52.9	81.9 (80.7–83.1)	91.2 (90.3–92.2)	91.3	81.8
3	4,569	1,920	42.0	72.5 (70.5–74.5)	94.3 (93.3–95.2)	90.2	82.5

AG-RDT: antigen rapid diagnostic test; CI: confidence interval; NPV: negative predictive value; PPV: positive predictive value; SARS-CoV-2: severe acute respiratory syndrome coronavirus 2; WHO: World Health Organization.

Data source: Czech Information System for Infectious Diseases.

The analyses presented in Tables 1–3 and Figures 1–2 were based on a dataset aggregating results for AG-RDT taken up to 3 days before the PCR under the assumption that a short interval between AG-RDT and PCR was indicative of a higher viral load while longer delay would be indicative of a low viral load. To test this assumption, we compared the average sensitivities determined in our study (Table 4) to those reported in the PEI study for high and low viral loads (unweighted average for all AG-RDTs evaluated by PEI [16]). For AG-RDTs recorded on the same day as the PCR test, we obtained the highest average sensitivity (80.4%). This was comparable to the average sensitivity for high viral load in the PEI evaluation (Cq ≤ 25) of 84.3%. The average sensitivity of 39.6% for AG-RTDs taken 3 days before the PCR was comparable to the average sensitivity of 40.1% for medium viral load (Cq > 25 – < 30) in the PEI study.

We saw the same pattern in the Pearson correlation coefficient between the computed sensitivities based on our data and the analytical sensitivities from the PEI study based on data for individual AG-RDTs. Table 5 shows that sensitivities determined on the high viral load sample pool by PEI correlated best with our data if restricted to PCR and AG-RDT taken on the same day. The highest correlation with medium viral loads as reported by PEI was obtained for the 3-day delay between AG-RDT and PCR in our data.

**Table 5:** Pearson correlation coefficient of sensitivities SARS-CoV-2 antigen rapid diagnostic tests between data from the Paul-Ehrlich Institute and this study, Czechia, 5 August–6 December 2021 (n = 24 test types)

Days between AG-RDT and PCR	0	1	2	3
Correlation with high viral load (Cq ≤ 25) in PEI results	0.16	0.13	0.07	0.05
Correlation with medium viral load (Cq > 25 – < 30) in PEI results	0.16	0.09	0.13	0.27

AG-RDT: antigen rapid diagnostic test; Cq: quantification cycle; PEI: Paul Ehrlich Institute; SARS-CoV-2: severe acute respiratory syndrome coronavirus 2.

Data source: Czech Information System for Infectious Diseases. The PEI list version used was 30 from May 2022. We included in the analysis a subset of 24 tests from Table 1 for which PEI sensitivities were available.

Note that the correlation for the high viral load was low, which can be partly explained by the fact that of 24 AG-RDTs from Table 1 for which the PEI study listed sensitivities, 11 had 100% sensitivity for high viral load according to PEI. This prevented a distinction between AG-RDT of different diagnostic performance because in our data, no AG-RDT obtained 100% sensitivity.

### Comparison with lists of approved AG-RDT published by public bodies

For this section, we used four lists of positively evaluated tests published by public bodies to create subsets of the tests listed in Table 1 and assessed their diagnostic performance. Our hypothesis was that AG-RDTs appearing on these lists will have higher sensitivity than tests not included on the respective list. As shown in Table 6, the sensitivity levels we obtained for AG-RDTs present on the WHO list, UK DHSC and EU Common lists were significantly higher than those of AG-RDTs not present on these lists (p < 0.05). The AG-RDTs on the WHO EUL (2022) had the highest average sensitivity levels. The second part of Table 4 shows that, regardless of the number of days between AG-RDT and PCR and of whether symptoms were reported or not, the AG-RDTs on the WHO 2022 list had higher sensitivity levels than AG-RDTs not on the list.

**Table 6:** Performance of SARS-CoV-2 antigen rapid diagnostic tests based on listing by public bodies, Czechia, 5 August–6 December 2021 (n = 38 test types)

Group of AG RDTs (distinct tests)	Total samples	PCR-positive cases	PCR test positivity in %	Sensitivity in % (95% CI)	Specificity in % (95% CI)
On WHO EUL 2020 (n = 2)	27,528	7,053	25.6	80.2 (79.3–81.2)	97.0 (96.8–97.3)
Not on WHO EUL 2020 (n = 36)	244,472	46,168	18.9	69.2 (68.8–69.6)	97.2 (97.1–97.2)
On WHO EUL 2022 list (n = 3)	34,353	9,002	26.2	81.3 (80.5–82.1)	96.7 (96.5–96.9)
Not on WHO EUL 2022 (n = 35)	237,647	44,219	18.6	68.5 (68.1–68.9)	97.2 (97.2–97.3)
On EU Common List (n = 20)	144,979	30,848	21.3	74.4 (73.9–74.9)	97.2 (97.1–97.3)
Not on EU Common List (n = 18)	127,021	22,377	17.6	65.5 (64.9–66.1)	97.1 (97.0–97.2)
On UK DHSC list (n = 7)	45,739	10,362	22.7	74.2 (73.3–75.0)	97.1 (96.9–97.2)
Not on UK DHSC list (n = 31)	226,261	42,859	18.9	69.8 (69.4–70.3)	97.2 (97.1–97.3)
On PEI list – passed sensitivity criteria (n = 20)	190,833	36,464	19.1	69.1 (68.6–69.6)	97.3 (97.2–97.3)
On PEI List – passed and on EU Common List (n = 15)	130,966	27,627	21.1	74.6 (74.1–75.1)	97.1 (97.0–97.2)
On PEI list – missing sensitivity criteria (n = 4)	51,664	9,700	18.8	74.6 (73.7–75.5)	96.8 (96.6–97.0)
Not on PEI list (n = 14)	29,503	7,057	23.9	73.3 (72.3–74.4)	97.2 (96.9–97.4)

AG-RDT: antigen rapid diagnostic test; CI: confidence interval; DHSC: Department of Health and Social Care; EU: European Union; EUL: Emergency Use List; PEI: Paul Ehrlich Institute; SARS-CoV-2: severe acute respiratory syndrome coronavirus 2; UK: United Kingdom; WHO: World Health Organization.

The numbers in parentheses after the sample types indicate the number of matching AG-RDTs from Table 1.

Data source: Czech Information System for Infectious Diseases.

Average sensitivity levels of tests that passed evaluation with PEI were unexpectedly lower than those of tests that did not pass. However, this effect disappeared when we excluded five tests that passed with PEI but were not present on the EU Common List. Of the four tests that did not pass with PEI, two also had sensitivity levels significantly below the average in our evaluation (tests no. 16 and 17; p < 0.05) but two had sensitivity levels significantly above the average (tests no. 34 and 36; p < 0.05).

## Discussion

The observed average sensitivity and specificity levels in our study were comparable to the values reported in a large meta-analysis by Brümmer et al. involving 133 studies and 61 different AG-RDTs [1]. We obtained the highest sensitivity level for Standard Q (SD Biosensor). A variation of the Standard Q test (Standard Q nasal) had the highest sensitivity among the instrument-free tests in that study [1]. Our sensitivity estimates for subgroups based on symptoms were higher than those reported [1] but the differences between the symptomatic and asymptomatic subgroups were similar. The lower sensitivity for the asymptomatic subgroup can be explained by higher Cq values in asymptomatic people [1] and AG-RDTs having lower sensitivity at higher Cq values [18].

We observed a statistically significantly lower AG-RDT sensitivity (p < 0.05) when testing children (0–12 years), which is consistent with other reports and has been attributed to lower SARS-CoV-2 viral load in children [24]. This pattern of lower sensitivity might also be attributed to a more problematic collection of samples, especially from smaller children. Sensitivity in adolescents was also found to be lower which can be explained by the SARS-CoV-2 viral load gradually increasing with age [25]. The observed decrease in sensitivity for low incidence and young age may be linked to specific AG-RDT types which do not reliably detect samples with lower viral load. One specific example was test no. 18 for which we recorded the biggest difference in sensitivity between the lowest and highest incidence. One variant of this test had zero sensitivity for all viral loads in the in vitro evaluation by PEI, while another version passed the PEI evaluation with 100.0% sensitivity for high viral loads (Cq ≤ 25) but had a sensitivity of 45.0% for medium viral load (Cq > 25 – < 30) [16].

According to both methods used to determine the sample type used with AG-RDTs, saliva tests were the least sensitive and nasopharyngeal swabs were the most sensitive. This is consistent with the technical report of the European Centre for Disease Prevention and Control, which states that the suitability of saliva sampling for rapid antigen tests is not supported by the available data and that nasopharyngeal sampling remains the gold standard [26]. Saliva sample type also had low sensitivity in some clinical and analytical studies [1,2], and the average viral load in saliva was reported to be lower than for nasopharyngeal swabs [27].

The AG-RDT test sensitivity in the vaccinated group was significantly higher than in the unvaccinated group, and subgroups based on presence or absence of symptoms showed the same pattern. This was contrary to the expectations – vaccinated persons are reported to have a lower viral load, which is associated with lower AG-RDT sensitivity [1,28]. The reasons for this difference are unclear and could be the subject of further investigation.

Our results show that AG-RDTs on the WHO EUL, the EU Common List or the UK’s DHSC list had significantly higher sensitivities than tests not present on these lists. The highest sensitivity was obtained for the WHO EUL 2022. While the sensitivity of AG-RDTs on the WHO EUL 2022 decreased for people without symptoms, this decrease was smaller than observed for AG-RTDs not on the WHO EUL 2022. This suggests that tests on these lists cover a wider Cq range and could be more suitable for testing scenarios characteristic of lower viral load.

Agreement of our results with the PEI study was less clear both on the aggregate and individual level. For the test most commonly used in Czechia according to our data (Lepu, no. 7), we determined an average sensitivity of 48.7%, while this test had 100.0% sensitivity in the evaluation by PEI for high viral load. This discrepancy was noted in at least one other study, which evaluated several versions of this test including different batches [2]. The authors observed *"very heterogeneous results for the Lepu medical AgPOCT*” and concluded that this *“precludes in our opinion the use of this product (or product family) for SARS-CoV-2 diagnostics”*.

Our study has several limitations. The sensitivity estimates could be affected by the fact that positive AG-RDTs were more likely to be followed by a confirmatory PCR than negative AG-RTDs, leading to underestimation of false-negative AG-RDTs. We did not stratify the analyses by presence of symptoms for individual AG-RDTs since there were few symptomatic cases per individual AG-RDT. For PCR within 3 days after the AG-RDTs, it is possible that some participants could have been infected between AG-RDT and PCR specimen collection. The strength of our conclusions on the low sensitivity of saliva tests is negatively affected by the low sensitivity of the most commonly used saliva test in the data set (no. 16), which accounted for most samples in the saliva category. In contrast, the second most commonly used saliva test (no. 21) had a sensitivity close to average. Future evolution of the ISIN database could involve the collection of Cq values, information on the sampling method and the batch of the AG-RDT. The latter could allow for the detection of AG-RDTs with unstable quality. For example, it has been shown that sensitivity of AG-RDTs can be lowered by inappropriate storage conditions such as high temperature [29]. It is possible that some AG-RDTs were used more often in the particular setting characteristic of higher (or lower) viral load, for example in hospitals as opposed to antigen testing sites in public locations. Therefore, the type of testing sites could be an additional useful explanatory variable for future analyses.

Regarding the applicability of results to other SARS-CoV-2 variants, Bekliz et al. suggest significantly lower sensitivity levels of AG-RDTs for the SARS-CoV-2 Omicron variant compared with Delta [30]. However, a regular analytical assessment of AG-RDTs carried out in Denmark did not confirm this: Of 28 evaluated tests, only five AG-RDTs had lower sensitivity for BA.1 than for Delta, while for BA.2, eight AG-RDTs had a higher sensitivity and only three had a lower sensitivity than for Delta [31]. Based on our preliminary data from ISIN from mid-January to February 2022, we also observed that AG-RDT sensitivities for the Omicron variant are generally slightly higher (data not shown).

## Conclusions

Our results show that AG-RDTs present on approved lists have on average higher sensitivity levels and may be more suitable for testing samples associated with lower viral load, e.g. for people with mild clinical signs or even without them. Furthermore, our study has shown that in vitro evaluations of AG-RTDs alone may not in all cases reliably identify high- and low-performing AG-RDTs, which may have implications for designing methodologies for validation of AG-RDTs. Data comparing the sensitivity and specificity of AG-RDTs for various subgroups may facilitate future cost–benefit analyses of AG-RDT testing. While following the decision of the EU Health Security Committee experts of 6 July 2021, AG-RDTs using only saliva samples were excluded from the EU Common List, they continued to be used in some EU countries including Czechia. Our results provide additional evidence that the use of some saliva-only AG-RDTs may not have been adequate for the SARS-CoV-2 Delta variant. This indication does not preclude the suitability of saliva samples for other SARS-CoV-2 variants such as Omicron.

## Supplementary Data

188000 Supplement
