# Supplementary material for: Role of population and test characteristics in antigen-based SARS-CoV-2 diagnosis, Czechia, August to November 2021
Source: Euro Surveill. 2022 Aug 18;27(33):2200070. doi: 10.2807/1560-7917.ES.2022.27.33.2200070 (PMC9389858; doi:10.2807/1560-7917.ES.2022.27.33.2200070)
Supplement: Supplement [file 22-00070_KLIEGR_Supplement.pdf]

This supplementary material is hosted by *Eurosurveillance* as supporting information alongside the article **Role of population and test characteristics in antigen-based SARS-CoV-2 diagnosis, Czechia, August 2021 to November 2021**, on behalf of the authors, who remain responsible for the accuracy and appropriateness of the content. The same standards for ethics, copyright, attributions and permissions as for the article apply. Supplements are not edited by *Eurosurveillance* and the journal is not responsible for the maintenance of any links or email addresses provided therein.

Appendix A. List of AG-RDTs in Table 1 by selected sample types as determined from the JRC COVID-19 In Vitro Diagnostic Devices and Test Methods Database. AG-RDTs for which there were multiple variants according to Appendix C were assigned to one of the sample type categories or excluded depending on the associated sample type(s) and the number of samples for each variant.

Test IDs	Sample type
**1,10,27,31,30**	Nasopharyngeal swab
**11,15,17,18,20,28,29**	Nasal swab
**2,3,6,8,9,13,19,32**	Nasal swab, Nasopharyngeal swab
**16**	Saliva

Appendix B. List of AG-RDTs by sample type as determined from test name from all AG-RDTs in the analysed dataset, excluding variations of test names and AG-RDT variants listed in Appendix C.

**Nasopharyngeal**
COVID-19 + Flu A&B + RSV Antigen Combo Rapid Test Cassette (Nasopharyngeal Swab) - Hangzhou Biotest Biotech Co., Ltd
COVID-19 Antigen Rapid Test (Nasopharyngeal Swab) - Acro Biotech Inc
COVID-19 and Influenza A+B Antigen Combo Rapid Test (Nasopharyngeal Swab) - Acro Biotech Inc
COVID-19 Antigen Rapid Test Cassette (Nasopharyngeal Swab) - Hangzhou Biotest Biotech Co., Ltd
COVID-19 Antigen Rapid Test (Nasopharyngeal Swab) - HANGZHOU ALLTEST BIOTECH CO., LTD.
Biomerica COVID-19 Antigen Rapid Test (nasopharyngeal swab) - Biomerica, Inc.
Dediatest 2019-nCoV Antigen Rapid Test (Nasopharyngeal) - Devidia GmbH
2019-nCoV Ag Rapid Detection Kit (Immuno-Chromatography) (Nasopharyngeal Swab) - Guangdong Longsee Biomedical Co., Ltd.
Rapid SARS-CoV-2 Antigen Test (nasopharyngeal specimen) - InTec PRODUCTS, INC.
COVID-19 Antigen Rapid Test (Nasopharyngeal Swab) - CITEST DIAGNOSTICS INC
COVID-19 + Flu A&B Antigen Combo Rapid Test Cassette (Nasopharyngeal Swab) - Hangzhou Biotest Biotech Co., Ltd
COVID-19 + Flu A&B + RSV + Adenovirus Antigen Combo Rapid Test Cassette (Nasopharyngeal Swab) - Hangzhou Biotest Biotech Co., Ltd
COVID-19 and Influenza A+B Antigen Combo Rapid Test (Nasopharyngeal Swab) - Ulti Med Products GmbH
COVID-19 Antigen Test Cassette (Nasopharyngeal Swab)(FIA) - HANGZHOU ALLTEST BIOTECH CO., LTD.
**Nasal**
SARS-CoV-2 Antigen Test Cassette (Nasal Swab Specimen) - Jiangsu Mole Bioscience Co., Ltd.
SARS-CoV-2 Rapid Antigen Test Nasal - Roche (SD BIOSENSOR)
AndLucky SARS CoV-2 Antigen Rapid Test, Anterior Nasal Check (minimal-invasiv) - Zhejiang Anji Sainfu Biotech Co., Ltd.
STANDARD Q COVID-19 Ag Test Nasal - SD Biosensor, Inc
SARS-CoV-2 Antigen Rapid Test (Nasal Swab) - HANGZHOU ALLTEST BIOTECH CO., LTD.

SARS-CoV-2 Antigen Self Test Nasal - Roche (SD BIOSENSOR)
BIOCREDIT COVID-19 Ag Test Nasal - RapiGEN Inc
SARS-CoV-2 Antigen Rapid Test Kit (Anterior Nasal - Zhejiang Anji Sainfu Biotech Co., Ltd.
SARS-CoV-2 Antigen Rapid Test (Nasal Swab) - Acro Biotech Inc
Immunobio SARS-CoV-2 Antigen ANTERIOR NASAL Rapid Test Kit (minimal invasive) - Hangzhou Immuno Biotech Co.,Ltd
FaStep COVID-19 Antigen Nasal Test Kit - Assure Tech (Hangzhou) Co., Ltd.
COVID-19 ANTIGEN RAPID TEST NASAL - Unioninvest Ltd.
ECOTEST COVID-19 Antigen Nasal Test Kit - Assure Tech (Hangzhou) Co., Ltd.
SARS-CoV-2 antigen IVD kit NASAL - Shenzhen Reagent Technology Co.,Ltd.
SARS-CoV-2 Antigen Test Kit (colloidal gold method) nasal swab - BIOTEKE CORPORATION (WUXI) CO., LTD
COVID-19 Antigen Rapid Test Cassette (Nasal Swab) - Hangzhou Biotest Biotech Co., Ltd
GSD NovaGen SARS-CoV-2 Ag Rapid Test (Nasal Swab) - NovaTec Immundiagnostica GmbH
GSD NovaGen SARS-CoV-2 Ag Rapid Test (Nasal Swab) - HANGZHOU ALLTEST BIOTECH CO., LTD.
AndLucky SARS-CoV-2 Antigen Anterior Nasal Rapid Test (minimal invasive) - Zhejiang Anji Sainfu Biotech Co., Ltd.
2019-nCoV Antigen Device (Anterior Nasal Swab) - Prestige Diagnostics
SARS-CoV-2 Antigen Test Kit (GICA)-Nasal (Anterioar) Swab - Shenzhen Kisshealth Biotechnology Co., Ltd
Ninonasal - NG Biotech
Accu-Tell COVID-19 Antigen Cassette (Nasal Swab) - AccuBioTech Co.,Ltd
2019-nCoV Antigen Device (Anterior Nasal Swab) - AMS UK (NI) Ltd
**Saliva**
Novel Coronavirus (SARS-Cov-2) Antigen rapid test Device (saliva) - Hangzhou Realy Tech Co., Ltd.
COVID-19 Antigen Rapid Test Kit (Saliva) - Safecare Biotech (Hangzhou) Co., Ltd.
Novel Coronavirus 2019-nCoV Antigen Test (Colloidal Gold) - Saliva - Beijing Hotgen Biotech Co., Ltd.
VivaDiag SARS-CoV-2 Ag Saliva Rapid Test - VivaChek Biotech (Hangzhou) Co., Ltd.
Saliva Orawell Covid_19 Ag - Jiangsu Well Biotech Co. Ltd
COVID-19 Antigen Rapid Test Cassette (Saliva) - Hangzhou Clongene Biotech Co., Ltd.
COVID-19 Antigen Saliva Test Kit - Assure Tech (Hangzhou) Co., Ltd.
COVID-19 Antigen Saliva Test kit (Colloidal Gold) - Nantong Diagnos Biotechnology Co.,Ltd.
SARS CoV 2 Ag Saliva Rapid Test - VivaChek Biotech (Hangzhou) Co., Ltd.
ACCEL ELISA COVID-19 Saliva Antigen Kit - Plexense, Inc.
SARS-CoV-2 antigen test kit (Saliva sample) - Shenzhen Dymind Biotechnology Co., Ltd
COVID-19 Salivary Antigen Rapid Test Kit (Colloidal Gold) - Jiangsu Konsung Bio-Medical Science And Technology Co., Ltd
COVID-19 Antigen Rapid Test Kit (Oral Saliva) - BEIJING KEWEI CLINICAL DIAGNOSTIC REAGENT INC.
2019-nCoV Ag Saliva Rapid Test Card (Immunochromatography) - Guangzhou Decheng Biotechnology Co., LTD
COVID-19 Ag Saliva Test Kit - Guangdong Wesail Biotech Co., Ltd.
COVID-19 Antigen Saliva Test Kit - Azure Biotech Inc.
AcuVid COVID-19 Rapid Antigen Saliva Test - Therma Bright Inc
SARS-CoV-2 Antigen Test Kit (Colloidal Gold) - Saliva - Hipro Biotechnology Co.,Ltd
2019-nCoV Saliva Ag EASY TEST - Guangzhou Decheng Biotechnology Co., LTD.
COVID-19 Antigen Saliva Test - AXIOM Gesellschaft für Diagnostica und Biochemica mbH
One Step Test for SARS-CoV-2 Antigen (Colloidal Gold) (Saliva) - Getein Biotech, Inc.

COVID-19 Saliva Ag Test - Core Technology Co., Ltd
Acura Speichel-Test Diagnos COVID-19 Antigen Saliva Test Kit - Acura Kliniken Baden-Baden GmbH
Accu-Tell COVID-19 Antigen Cassette (Saliva) - AccuBioTech Co.,Ltd
GLINE-2019-nCoV Ag Saliva - SHENZHEN YHLO BIOTECH CO., Ltd
Coronavirus (SARS-Cov-2) Antigen Rapid Test Device (Saliva) - Innovation Biotech (Beijing) Co.,ltd
SARS-CoV-2 Antigen Rapid Test (Saliva) - Xiamen Wiz Biotech Co., Ltd
EONbtTM COVID-19 Antigen Saliva Detection Kit - Eon Biotechnology Limited
TGS Velox Ag COVID-19 POCT SALIVA (25 test) - Technogenetics s.r.l.
New Coronavirus (COVID-19) Antigen Rapid Test (saliva) - Anhui Formaster Biosci Co., Ltd.
Unibioscience Covid-19 Rapid Antigen Saliva Test - Unibioscience AS
Saliva SARS-Cov-2(2019-nCoV) Antigen Combined Test Kit (Nanocarbon Assay) - Ningbo Beautiful Life Medical Biotechnology Development Co., Ltd.
COVDIAG SALIVA - TODA PHARMA
LollyTest 2019-CoV Ag Saliva Rapid Test Card - Hunan Zonka Biotech Co., Ltd
SARS-CoV-2 Antigen Test Kit (colloidal gold method) saliva - BIOTEKE CORPORATION (WUXI) CO., LTD

Appendix C. Other variants of AG-RDTs included under one entry in Table 1**.**

id	Additional AG-RDT variants
7	2019-nCoV Antigen Rapid Test Kit (Colloidal Gold Immunochromatography)
13	2019-nCoV Ag Rapid Test Kit<br>V-CHEK, 2019-nCoV Ag Rapid Test Kit
17	Novel Coronavirus (SARS-Cov-2) Antigen Rapid Test Device (nasal swab)
18	COVID-19 Test Kit (Colloidal Gold Method)<br>COVID-19 Test Kit (Colloidal Gold Method)(Nasal Swab)
21	Orawell COVID-19 Ag Rapid Saliva Test Device (CO-03)<br>Orawell COVID-19 Ag Rapid Saliva Test Device (CO-07)<br>Orawell COVID-19 Ag Rapid Saliva Test Device CO-07(One Step)
37	SARS-CoV-2 Antigen Rapid Test Kit (Colloidal gold Immunoassay)
